# Neutrophil-to-lymphocyte ratio is associated with in-hospital mortality in patients with cirrhosis and pneumonia: a multicenter retrospective study

**DOI:** 10.3389/fmed.2026.1963013

**Published:** 2026-10-19

**Authors:** Hui Zhang, Lixia Huang, Pengpeng Sun, Youling Dong, Ye Gu, Xian Chen, Shuang Suo, Meng Zhang, Ye Zhang

**Affiliations:** 1 Department of Critical Care Medicine, Qingdao Central Hospital, University of Health and Rehabilitation Sciences, Qingdao, China; 2 Department of Respiratory, Beijing Jingmei Group General Hospital, Beijing, China; 3 Department of Emergency, Chuiyangliu Hospital Affiliated to Tsinghua University, Beijing, China; 4 Department of Emergency, Chinese People’s Armed Police Force Characteristic Medical Center, Tianjin, China; 5 Department of Respiratory and Critical Care Medicine, Beijing Mentougou District Hospital, Beijing, China; 6 Beijing Tongren Hospital Mentougou Campus, Capital Medical University, Beijing, China; 7 Department of Respiratory, Beijing You’an Hospital, Capital Medical University, Beijing, China; 8 Department of Critical Care Medicine, Beijing Mentougou District Hospital, Beijing, China

**Keywords:** cirrhosis, in-hospital mortality, neutrophil-to-lymphocyte ratio, pneumonia, prognosis

## Abstract

**Background:**

Patients with cirrhosis complicated by pneumonia face high in-hospital mortality, yet reliable prognostic tools remain limited. The neutrophil-to-lymphocyte ratio (NLR) is a readily available inflammatory marker with established prognostic value across various diseases. However, multicenter evidence on the independent association of log_10_NLR with mortality in this specific population is lacking. This study therefore investigated the association between log_10_NLR and in-hospital mortality in patients with cirrhosis and pneumonia, and evaluated its discriminative performance.

**Methods:**

This multicenter study included 851 patients with cirrhosis and pneumonia from five Chinese centers (2016–2025). Admission log_10_NLR was the exposure and in-hospital mortality the outcome. Multivariable logistic regression with multiple imputation, restricted cubic splines, and ROC analysis was used; the incremental value of log_10_NLR beyond the Child-Pugh and CURB-65 scores was assessed by the DeLong test, calibration curves, and decision-curve analysis.

**Results:**

Among 851 patients, in-hospital mortality was 14.9%. In the fully adjusted model, a 1-unit increase in log_10_NLR (a 10-fold increase in NLR) was associated with higher odds of in-hospital death (OR: 3.82, 95% CI: 1.97–7.38; *P* < 0.001). Across tertiles, the odds increased progressively (second: OR: 2.81, 95% CI: 1.44–5.47; third: OR: 3.35, 95% CI: 1.65–6.78; *P* for trend = 0.001), and restricted cubic spline analysis showed a linear relationship (*P* for overall association = 0.003; *P* for non-linearity = 0.431). The AUC for log_10_NLR was 0.707 (95% CI: 0.660–0.754), numerically higher than for Child-Pugh (0.647) and CURB-65 (0.658), though not significantly (DeLong *P* > 0.05). Adding log_10_NLR to each score significantly improved discrimination (ΔAUC = 0.090 and 0.076; both *P* < 0.001) and net benefit. An exploratory cutoff of approximately 5 (sensitivity 74.0%, specificity 57.6%) requires independent validation.

**Conclusions:**

Log_10_NLR was independently associated with in-hospital mortality in cirrhosis with pneumonia, with a linear dose–response relationship. Its discrimination alone was comparable to the Child-Pugh and CURB-65 scores; however, adding log_10_NLR significantly improved discrimination (ΔAUC = 0.090 and 0.076; both *P* < 0.001) and net benefit, suggesting a complementary role in risk stratification.

## Introduction

Cirrhosis is the end-stage of various chronic liver diseases. Globally, there are approximately 112 million patients with compensated cirrhosis and 10.6 million with decompensated cirrhosis, the risk of death for these two groups is 5 and 10 times higher, respectively, than that of the general population (1), imposing a heavy disease burden. Patients with cirrhosis exhibit significant immune dysfunction (2), and the incidence of infections and sepsis is markedly elevated. Pneumonia is a common type of infection among patients with cirrhosis. The CLEARED international multicenter study and a prospective cohort study in Mexico have reported that pneumonia accounts for 17.2%–28.2% of infectious complications among hospitalized patients with cirrhosis (3, 4). In addition to triggering hepatic encephalopathy, pneumonia often progresses to acute kidney injury and acute-on-chronic liver failure (ACLF), significantly increasing the risk of removal from the liver transplant waiting list and the risk of death (5). Viasus et al. (6) reported that patients with cirrhosis complicated by community-acquired pneumonia had more severe disease and higher mortality rates; although the MELD-CAP score they developed (combining MELD, multilobar infiltration, and septic shock) demonstrated superior predictive performance compared to traditional scores, it involves numerous parameters and is complex to calculate. For such high-risk patients, simple and reliable biomarkers may facilitate early risk identification and guide clinical interventions.

The neutrophil-to-lymphocyte ratio (NLR) is the ratio of the number of neutrophils to the number of lymphocytes in peripheral blood, has been extensively studied in the prognosis of both cirrhosis and pneumonia (7, 8). Neutrophils are the primary effector cells of the innate immune system (9) and increase significantly in peripheral blood early during infection; lymphocytes are the core cells of the adaptive immune system (10), and during infection, inflammatory factors induce their apoptosis, decreasing their peripheral blood count. Patients with cirrhosis exhibit underlying immune dysfunction, persistent inflammation, and splenic dysfunction, as well as an imbalance between neutrophils and lymphocytes (2). When pneumonia occurs, the systemic inflammatory response is further exacerbated, stimulating the release of neutrophils; at the same time, inflammatory cytokines such as tumor necrosis factor-α and interleukin-6 accelerate lymphocyte apoptosis, leading to a more significant increase in the NLR (10). Previous studies have shown that the NLR is independently associated with decompensation and mortality in hospitalized patients with cirrhosis (11), and its predictive ability is independent of the MELD (12) and Child-Pugh (7) scores. The predictive value of NLR for in-hospital mortality in patients with pneumonia has also been confirmed (13), and it is positively correlated with the CURB-65 score (14). To date, there have been few studies examining the prognostic significance of the NLR in patients with cirrhosis complicated by pneumonia. This study aims to evaluate the predictive value of the NLR in this patient population and compare it with traditional scoring systems [Child-Pugh (15), CURB-65 (16)].

## Materials and methods

This study was conducted in accordance with the principles of the Declaration of Helsinki and was approved by the Institutional Review Board of Beijing Mentougou District Hospital (Approval Number: 2025-QYYPJ-A01). Additionally, the study was registered with the Chinese Clinical Trial Registry in February 2025 (Registration Number: ChiCTR2500097772). Given the retrospective nature of the study, the requirement for obtaining informed consent was waived by the Institutional Review Board of Beijing Mentougou District Hospital.

Peripheral neutrophil and lymphocyte counts were measured by standardized automated blood cell analyzers at each participating center. Despite inherent inter-laboratory variability in multicenter retrospective research, all laboratories complied with certified national quality control standards for blood routine assays.

### Study population and data collection

Data were retrieved from electronic medical records. The study population included patients aged ≥18 years with concurrent cirrhosis and pneumonia confirmed by clinical, laboratory, or radiological evidence. Exclusion criteria comprised age <18 years, absent or unconfirmed diagnostic proof, missing data for the primary endpoint (in-hospital mortality), and unavailable admission neutrophil or lymphocyte counts; only the initial admission record was retained for patients with repeated hospitalizations.

Collected variables encompassed demographic characteristics (age, sex); comorbid conditions [history of malignancy, coronary atherosclerotic heart disease (CAD), hypertension, diabetes mellitus (DM), heart failure (HF), and hepatitis etiology (viral, alcoholic, other, or none)]; baseline admission laboratory parameters, including white blood cell count, neutrophil count, lymphocyte count, red blood cell count (RBC), hemoglobin (Hb), platelet count (PLT), alanine transaminase (ALT), aspartate transaminase (AST), total bilirubin (TBIL), direct bilirubin (DBIL), albumin (ALB), blood urea nitrogen (BUN), creatinine (Cr), blood glucose (GLU), prothrombin time (PT), activated partial thromboplastin time (APTT), international normalized ratio (INR), thrombin time (TT), and D-dimer; alongside calculated disease severity scores [Child-Turcotte-Pugh (CTP) score and CURB-65 score].

### Variable definitions

Before enrollment, all patients had a diagnosis of cirrhosis established by a hepatologist or gastroenterologist, based on clinical, radiological, endoscopic, elastographic, and/or histopathological evidence, or on a documented history of cirrhosis in the medical record, consistent with current national and international guidelines (17, 18). The diagnosis of pneumonia requires simultaneous fulfillment of imaging and clinical laboratory criteria: new pulmonary infiltrates on chest radiograph or computed tomography (CT); and at least one of the following clinical manifestations: new or worsening cough, expectoration, fever (oral temperature ≥37.3 °C), signs of lung consolidation or rales, or abnormal peripheral white blood cell count. The neutrophil-to-lymphocyte ratio (NLR) is calculated as the peripheral blood neutrophil count (×10^9^/L) divided by the peripheral blood lymphocyte count (×10^9^/L).

The outcome of this study was in-hospital mortality. Patients who were discharged home were considered as survivors during hospitalization.

The Child–Turcotte–Pugh (CTP) score was assessed based on five indicators with 1–3 points for each item: hepatic encephalopathy (1 point for no encephalopathy, 2 points for grade 1–2 encephalopathy, 3 points for grade 3–4 encephalopathy), ascites (1 point for absent ascites, 2 points for diuretic-responsive ascites, 3 points for refractory or moderate-to-severe ascites), total bilirubin (μmol/L; 1 point for <34.2, 2 points for 34.2–51.3, 3 points for >51.3), serum albumin (g/L; 1 point for >35, 2 points for 28–35, 3 points for <28), and international normalized ratio (INR; 1 point for <1.7, 2 points for 1.7–2.3, 3 points for >2.3). All enrolled patients were divided into Child–Pugh class A (total score: 5–6), class B (total score: 7–9) and class C (total score: 10–15) (15). The CURB-65 scoring system incorporated five clinical variables, and 1 point was given whenever a criterion was satisfied: new confusion manifesting as disorientation to person, place, or time, serum urea concentration >7 mmol/L, respiratory rate ≥30 breaths/min, hypotension defined as systolic blood pressure <90 mmHg or diastolic blood pressure ≤60 mmHg, and age ≥65 years. The aggregate score ranged from 0 to 5. (19).

### Statistical analysis

Histogram plots and the Shapiro–Wilk test revealed a marked right skew in raw NLR values (*P* < 0.001). Because NLR showed a markedly right-skewed distribution, it was log_10_-transformed to reduce the influence of extreme values (20) and improve the functional relationship between NLR and the log odds of in-hospital mortality. Accordingly, log_10_ transformation was performed: log_10_NLR = log_10_(neutrophil count/lymphocyte count). Baseline clinical characteristics were stratified and presented by tertiles of log_10_NLR. Normally distributed continuous variables were expressed as mean ± standard deviation (SD) and compared using one-way analysis of variance (ANOVA); skewed continuous variables were presented as median (interquartile range, IQR) and compared using the Kruskal–Wallis test. Categorical variables were expressed as frequencies (percentages) and compared using the chi-square test or Fisher's exact test, as appropriate.

Multivariable logistic regression was used to evaluate the association between log_10_NLR and in-hospital mortality. Four models with progressive covariate adjustment were constructed: Model 1, age and sex; Model 2, Model 1 plus CAD, hypertension, DM, hepatitis type, and hospital cohort; Model 3, Model 2 plus Child-Pugh and CURB-65 scores; and Model 4 (fully adjusted), Model 2 plus blood urea nitrogen, WBC, albumin, and INR. Covariate selection was based on univariable analysis (*P* < 0.05) and clinical relevance. Pairwise correlations among candidate predictors were visualized using a correlation matrix and heatmap (Supplementary Figures S1, S2). Multicollinearity was further quantified using variance inflation factor (VIF), and variables with VIF > 5 were excluded. To examine the dose-response relationship between log_10_NLR and mortality risk, the median value of each log_10_NLR group was used as a continuous variable for trend analysis. Restricted cubic spline (RCS) regression with 4 knots was used to examine the dose-response relationship between log_10_NLR and in-hospital mortality. The reference value was set at the median log_10_NLR (0.6682) of the entire cohort. Models were adjusted for age, sex, CAD, hypertension, DM, hepatitis types, hospital cohorts, Urea, WBC, INR, and ALB. Sensitivity analyses were performed to verify the robustness of the main findings: (1) a complete-case analysis was conducted by excluding any sample with missing values (*n* = 392); (2) To account for clustering of patients within hospitals, we additionally fitted a generalized linear mixed-effects logistic regression model with a random intercept for participating center, including the same covariates as the fully adjusted model. Between-center heterogeneity was quantified by the random-intercept variance and the intraclass correlation coefficient (ICC). (3) Given the long study period, we performed sensitivity analyses additionally adjusting for calendar time, modeled both as a categorical variable (admission period: 2016–2021 vs. 2022–2025) and as a continuous variable (admission year). Effect modification by calendar period was assessed using an interaction term. Subgroup analyses were conducted across different strata, and interaction tests were performed. Receiver operating characteristic (ROC) curve analysis was used to evaluate the discriminative performance of log_10_NLR, the Child-Pugh score, and the CURB-65 score, and the DeLong test was used for pairwise comparisons of AUCs. The optimal cutoff value for log_10_NLR was determined by maximizing the Youden index (sensitivity + specificity − 1). To assess whether log_10_NLR provides prognostic information beyond established scores, we compared the AUC of Child-Pugh alone vs. Child-Pugh + log_10_NLR, and of CURB-65 alone vs. CURB-65 + log_10_NLR, using the DeLong test, with ΔAUC reported for each comparison. Model calibration was evaluated using calibration curves, and clinical utility using decision-curve analysis (DCA) across a range of clinically relevant threshold probabilities.

This study was conducted in strict accordance with the STROBE guidelines (21). Missing data are summarized in Supplementary Table S1. Multiple imputation by chained equations (MICE) was performed using the mice package in R 4.2.2 (22). The imputation model included all analysis variables together with auxiliary variables associated with missingness; the exposure (log_10_NLR) and the outcome (in-hospital mortality) had no missing values and were therefore included as predictors rather than imputed. We systematically compared the baseline clinical data between complete-case subjects and subjects with missing cases (Supplementary Table S7). The results showed that most core clinical and laboratory indicators had no statistically significant differences between the two groups of study subjects. This suggests that the missing-data mechanism in this study approximates missing at random (MAR), which satisfies the prerequisite for multiple imputation and serves as the main rationale for our adoption of MICE multiple imputation. Variables with missing data were imputed using variable-specific models: predictive mean matching for continuous variables, and logistic, multinomial logistic, or proportional-odds models for categorical variables. Individual score components (e.g., ascites, hepatic encephalopathy, and disturbance of consciousness) were imputed, and Child-Pugh and CURB-65 scores were recomputed after imputation.

The primary analysis used *m* = 5 imputations; because missingness reached 38% for some variables, a sensitivity analysis with *m* = 40 imputations was also performed and yielded essentially identical results (Supplementary Table S2). Estimates were combined across imputed datasets using Rubin's rules (23). Because the exposure and the outcome were complete and no covariate in the fully adjusted model had more than 4.0% missing values (INR 4.0%; all other covariates ≤1.8%), the fraction of missing information for the log_10_NLR coefficient is small; the criterion that the number of imputations should be at least 100 times the fraction of missing information is therefore satisfied by *m* = 5 (24). Convergence was assessed using trace plots of the mean and standard deviation of imputed values across iterations, which showed no systematic trend (Supplementary Figure S4).

Sensitivity analysis using complete cases (*n* = 392) confirmed the robustness of the primary findings (Supplementary Table S3). All statistical analyses were conducted using R 4.2.2 and Free Statistics 2.5.1 software. Statistical significance was defined as a two-sided *P*-value <0.05.

## Results

### Baseline characteristics

A total of 851 patients with liver cirrhosis complicated by pneumonia from 5 medical centers were enrolled in this study (Figure 1); all primary analyses were based on the multiply imputed dataset (*n* = 851), and complete-case analyses (*n* = 392) are reported as sensitivity analyses. The baseline characteristics of the study population are presented in Table 1. The mean age was 66.5 ± 12.7 years, and 63% of patients were male. Patients were stratified into three groups according to tertiles of log_10_NLR: T1 (*n* = 284), T2 (*n* = 283), and T3 (*n* = 284). The median NLR values were 2.1 (IQR: 1.6–2.6), 4.7 (IQR: 3.8–5.9), and 12.3 (IQR: 8.9–19.3) for the T1, T2, and T3 groups, respectively. There were no statistically significant differences in sex, age, comorbidities (coronary atherosclerotic heart disease, hypertension, diabetes mellitus), and hepatitis type among the three groups (*P* > 0.05). The prevalence of malignant tumor history was significantly higher in the T3 (27.8%) group compared with the T1 (19.4%) and T2 (15.9%) groups (*P* = 0.002).

**Figure 1:**
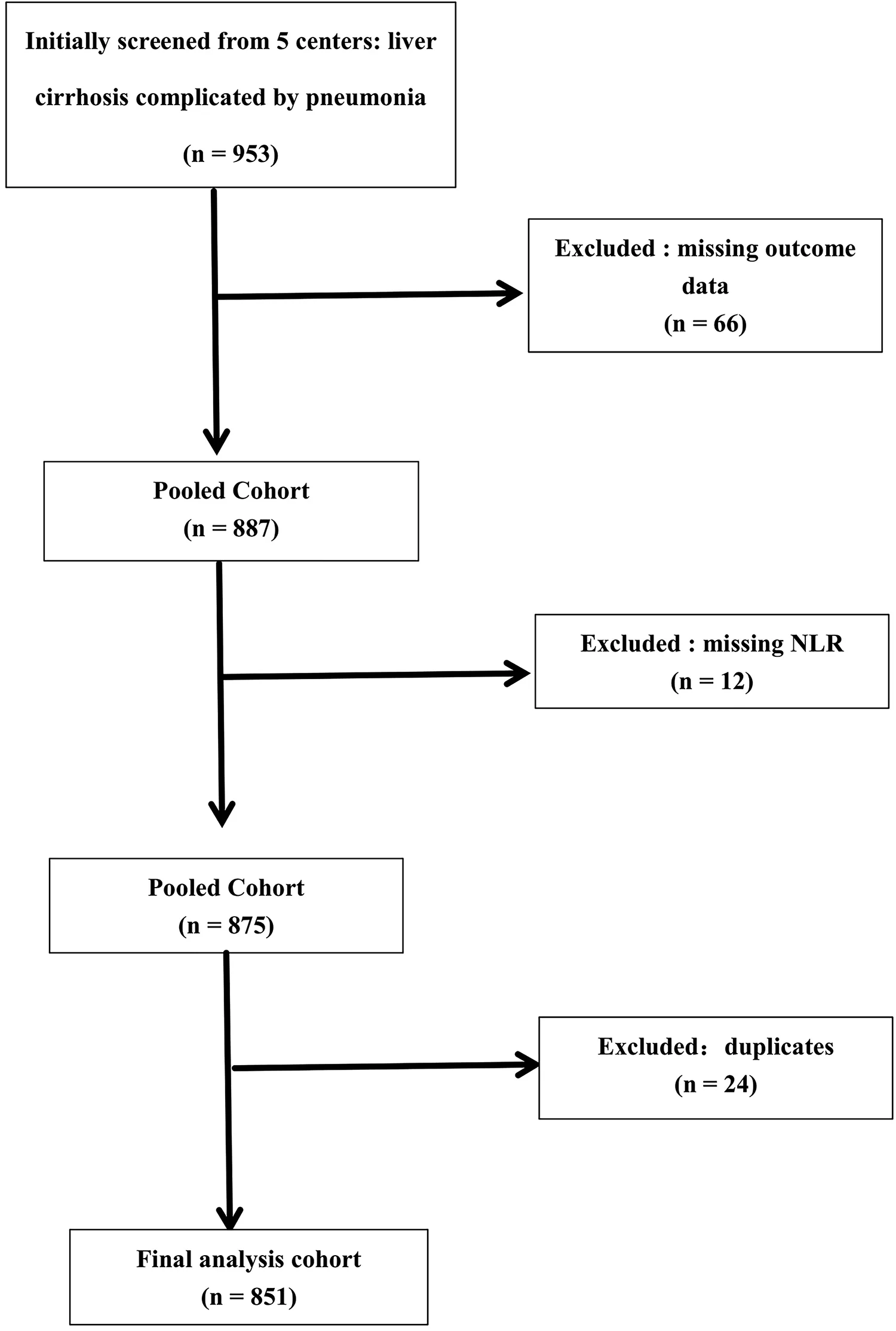
Flowchart of the study sample. NLR: neutrophil-to-lymphocyte ratio.

**Table 1:** Baseline characteristics.

Variables	Total (*n* = 851)	log_10_NLR			*P*-value
		T1 (*n* = 284) T2 (*n* = 283) T3 (*n* = 284)			
NLR^b^	4.7 (2.6, 8.9)	2.1 (1.6, 2.6)	4.7 (3.8, 5.9)	12.3 (8.9, 19.3)	< 0.001
Male^c^	536 (63.0)	179 (63)	172 (60.8)	185 (65.1)	0.56
Age^a^, years	66.5 ± 12.7	66.2 ± 12.2	66.7 ± 12.4	66.6 ± 13.5	0.901
WBC^b^ (×10^9^/L)	5.6 (3.7, 8.5)	4.3 (3.0, 5.8)	5.3 (3.7, 7.7)	8.5 (5.7, 12.7)	< 0.001
Hb^a^ (g/L)	101.9 ± 29.6	107.8 ± 29.8	99.9 ± 29.5	98.0 ± 28.9	< 0.001
PLT^b^ (×10^9^/L)	98.0 (64.0, 148.0)	97.0 (65.8, 144.0)	99.0 (65.0, 140.0)	99.0 (60.8, 159.2)	0.918
ALT^b^ (U/L)	21.7 (14.0, 35.2)	23.0 (14.9, 33.2)	21.0 (13.7, 32.5)	21.7 (14.0, 38.7)	0.163
AST^b^ (U/L)	34.1 (22.4, 56.4)	34.5 (23.0, 55.0)	33.0 (21.4, 53.0)	35.5 (22.0, 61.1)	0.462
TBIL^b^ (μmol/L)	23.0 (13.7, 42.1)	20.4 (13.4, 36.5)	23.0 (13.0, 41.3)	27.4 (15.4, 55.9)	0.003
DBIL^b^(μmol/L)	10.5 (5.7, 22.0)	8.6 (5.2, 17.2)	10.8 (5.9, 18.6)	12.9 (6.4, 30.2)	< 0.001
ALB^a^ (g/L)	31.1 ± 5.9	32.6 ± 6.2	31.3 ± 5.5	29.5 ± 5.7	< 0.001
Urea^b^ (mmol/L)	6.8 (4.8, 11.1)	5.5 (4.2, 7.7)	6.7 (4.9, 10.2)	9.1 (5.9, 16.6)	< 0.001
Cr^b^(μmol/L)	72.0 (55.0, 102.0)	65.9 (52.8, 90.5)	72.0 (56.0, 97.0)	81.5 (59.0, 132.2)	< 0.001
GLU^a^ (mmol/L)	7.2 ± 3.5	6.5 ± 3.0	7.3 ± 3.6	7.8 ± 3.7	< 0.001
PT^a^ (s)	16.0 ± 6.5	15.0 ± 4.0	15.4 ± 3.4	17.6 ± 9.8	< 0.001
APTT^a^ (s)	37.9 ± 10.0	37.7 ± 9.8	36.7 ± 8.3	39.3 ± 11.4	0.007
INR^a^	1.4 ± 0.7	1.3 ± 0.4	1.3 ± 0.3	1.5 ± 1.0	< 0.001
TT^a^ (s)	18.4 ± 3.3	18.7 ± 3.3	18.4 ± 3.2	18.2 ± 3.3	0.202
D-dimer^b^ (mg/L)	2.2 (0.8, 5.4)	1.6 (0.6, 4.5)	2.2 (0.8, 5.0)	2.9 (1.3, 6.7)	< 0.001
CAD^c^	168 (19.7)	52 (18.3)	67 (23.7)	49 (17.3)	0.12
Hypertension^c^	354 (41.6)	115 (40.5)	115 (40.6)	124 (43.7)	0.688
DM^c^	248 (29.1)	74 (26.1)	80 (28.3)	94 (33.1)	0.168
Heart failure^c^	64 (7.5)	14 (4.9)	21 (7.4)	29 (10.2)	0.058
Malignant tumor history^c^	179 (21.0)	55 (19.4)	45 (15.9)	79 (27.8)	0.002
Hepatitis^c^					0.373
None	206 (24.2)	60 (21.1)	74 (26.1)	72 (25.4)	
Viral	307 (36.1)	107 (37.7)	95 (33.6)	105 (37.0)	
Alcoholic	154 (18.1)	50 (17.6)	60 (21.2)	44 (15.5)	
Others	184 (21.6)	67 (23.6)	54 (19.1)	63 (22.2)	
Child-Turcotte-Pugh(CTP)^a^ score	7.7 ± 2.1	7.3 ± 2.0	7.5 ± 1.9	8.4 ± 2.2	< 0.001
CTP classification^c^					< 0.001
A	283 (33.3)	123 (43.3)	104 (36.7)	56 (19.7)	
B	412 (48.4)	126 (44.4)	140 (49.5)	146 (51.4)	
C	156 (18.3)	35 (12.3)	39 (13.8)	82 (28.9)	
CURB-65 score^c^					< 0.001
<3	703 (82.6)	253 (89.1)	239 (84.5)	211 (74.3)	
≥3	148 (17.4)	31 (10.9)	44 (15.5)	73 (25.7)	
Non-survival^c^	127 (14.9)	15 (5.3)	43 (15.2)	69 (24.3)	< 0.001
Hospital.cohorts^c^					
Mentougou	198 (23.3)	67 (23.6)	69 (24.4)	62 (21.8)	0.968
Armed Police	181 (21.3)	60 (21.1)	59 (20.8)	62 (21.8)	
Qingdao	105 (12.3)	34 (12)	38 (13.4)	33 (11.6)	
Jingmei Group	180 (21.2)	65 (22.9)	55 (19.4)	60 (21.1)	
Chuiyangliu	187 (22.0)	58 (20.4)	62 (21.9)	67 (23.6)	
Admitted.to.ICU^c^					< 0.001
No	708 (83.2)	259 (91.2)	234 (82.7)	215 (75.7)	
Yes	143 (16.8)	25 (8.8)	49 (17.3)	69 (24.3)	

SD, standard deviation; log_10_NLR, logarithm base 10 of the neutrophil-to-lymphocyte ratio; WBC, white blood cell count; RBC, red blood cell count; Hb, hemoglobin; PLT, platelet count; ALT, alanine aminotransferase; AST, aspartate aminotransferase; TBIL, Total bilirubin; DBIL, direct bilirubin; ALB, albumin; Cr, serum creatinine; GLU, glucose; PT, prothrombin time; APTT, activated partial thromboplastin time; INR, international normalized ratio; TT, thrombin time; CAD, Coronary Atherosclerotic Heart Disease; DM, Diabetes Mellitus.

a Data are presented as mean ± SD.

b Median (IQR).

c Or *n* (%).

Compared with patients in the T1 group, those in the T3 group had significantly higher white blood cell counts, lower hemoglobin concentrations, poorer liver function (lower albumin levels and higher bilirubin levels), worse renal function (higher urea and creatinine levels), and coagulation abnormalities (prolonged prothrombin time and elevated D-dimer levels).

The prevalence of Child-Pugh class C was markedly higher in the T3 group (28.9%) compared with the T1 group (12.3%, *P* < 0.001), as was the proportion of patients with CURB-65 scores ≥3 (25.7% vs. 10.9%, *P* < 0.001). The in-hospital mortality rate differed significantly among the three groups (*P* < 0.001). The mortality rate was highest in the T3 group (24.3%), followed by T2 (15.2%) and T1 (5.3%), suggesting a dose-response relationship between log_10_NLR levels and in-hospital mortality risk. Across the five participating centers, the numbers of enrolled patients were 198 (23.3%) at Mentougou, 181 (21.3%) at Armed Police, 105 (12.3%) at Qingdao, 180 (21.2%) at Jingmei Group, and 187 (22.0%) at Chuiyangliu. The distribution of patients across the log_10_NLR tertiles did not differ significantly among centers (*P* = 0.968), indicating that the exposure was broadly balanced across the participating hospitals. Among all 851 patients, 143 (16.8%) were transferred to the ICU while 708 (83.2%) were not. Stratified by log_10_NLR tertiles, the ICU transfer rates for T1, T2 and T3 groups were 8.8%, 17.3% and 24.3%, respectively. The proportion of ICU transfer varied significantly across tertile groups (*P* < 0.001), showing a significant rising trend of ICU transfer rate as log_10_NLR increased (*P* < 0.001).

### Association between log_10_NLR and in-hospital mortality in patients with liver cirrhosis complicated by pneumonia

In total, 851 eligible patients from five participating centers were included in this study. Supplementary Table S4 summarizes the distribution of patients and the in-hospital mortality rate across centers. The overall in-hospital mortality was 14.9% (127/851). Table 2 presents the results of multivariable logistic regression analysis examining the association between log_10_NLR and in-hospital mortality. When log_10_NLR was included as a continuous variable, all models demonstrated that each unit increase in log_10_NLR was significantly associated with higher odds of in-hospital mortality: crude OR: 5.74 (95% CI: 3.55–9.30, *P* < 0.001). After adjustment for potential confounders, this association remained stable (Model 4: OR: 3.82, 95% CI: 1.97–7.38, *P* < 0.001). For clinical interpretation, each doubling of NLR was associated with 1.50-fold higher odds of in-hospital death (OR = 1.50, 95% CI: 1.23–1.83, *P* < 0.001). When log_10_NLR was analyzed as tertiles, with the first tertile (T1) as the reference, the odds of in-hospital mortality increased monotonically across tertiles. In the fully adjusted model (Model 4), patients in the third tertile (T3) had significantly higher odds of in-hospital death (OR: 3.35, 95% CI: 1.65–6.78; *P* = 0.001), and those in the second tertile (T2) also had significantly higher odds (OR: 2.81, 95% CI: 1.44–5.47; *P* = 0.002). A significant test for trend (*P* for trend = 0.001) supported a dose–response association between admission log_10_NLR and in-hospital mortality.

**Table 2:** Multivariable logistic regression analysis of log_10_NLR and in-hospital mortality.

Variable	Crude		Model 1		Model 2		Model 3		Model 4	
	OR	*P-*value	OR	*P-*value	OR	*P*-value	OR	*P*-value	OR	*P*-value
	(95%CI)		(95%CI)		(95%CI)		(95%CI)		(95%CI)	
Log_10_NLR	5.74 (3.55–9.3)	<0.001	5.61 (3.45–9.11)	<0.001	7.69 (4.43–13.36)	<0.001	5.14 (2.88–9.19)	<0.001	3.82 (1.97–7.38)	<0.001
log_10_NLR										
T1 (≤0.495)	1(Ref)		1(Ref)		1(Ref)		1(Ref)		1(Ref)	
T2 (> 0.495 to ≤ 0.860)	3.21 (1.74–5.93)	<0.001	3.22 (1.74–5.95)	<0.001	3.43 (1.8–6.56)	<0.001	3.49 (1.78–6.88)	<0.001	2.81 (1.44–5.47)	0.002
T3 (>0.860)	5.76 (3.2–10.35)	<0.001	5.76 (3.2–10.38)	<0.001	7.35 (3.91–13.79)	<0.001	5.08 (2.62–9.88)	<0.001	3.35 (1.65–6.78)	0.001
*P* for trend	< 0.001		<0.001		<0.001		<0.001		0.001	

OR, odds ratio; CI, confidence interval; log_10_NLR, logarithm base 10 of the neutrophil-to- lymphocyte ratio; CAD, Coronary Atherosclerotic Heart Disease; DM, Diabetes Mellitus; WBC, white blood cell count; ALB, albumin; INR, international normalized ratio.

Model 1: adjusted for age, sex.

Model 2: adjusted for age, sex, CAD, hypertension, DM, hepatitis types, hospital cohorts.

Model 3: adjusted for age, sex, CAD, hypertension, DM, hepatitis types, hospital cohorts, Child-Pugh, CURB-65.

Model 4: adjusted for age, sex, CAD, hypertension, DM, hepatitis types, hospital cohorts, Urea, WBC, INR, ALB.

Restricted cubic spline analysis demonstrated a significant association between log_10_NLR and in-hospital mortality (overall *P* = 0.003), with no statistically significant evidence of nonlinearity (nonlinear *P* = 0.431) (Figure 2). Log_10_NLR was associated with in-hospital mortality in a linear fashion.

**Figure 2:**
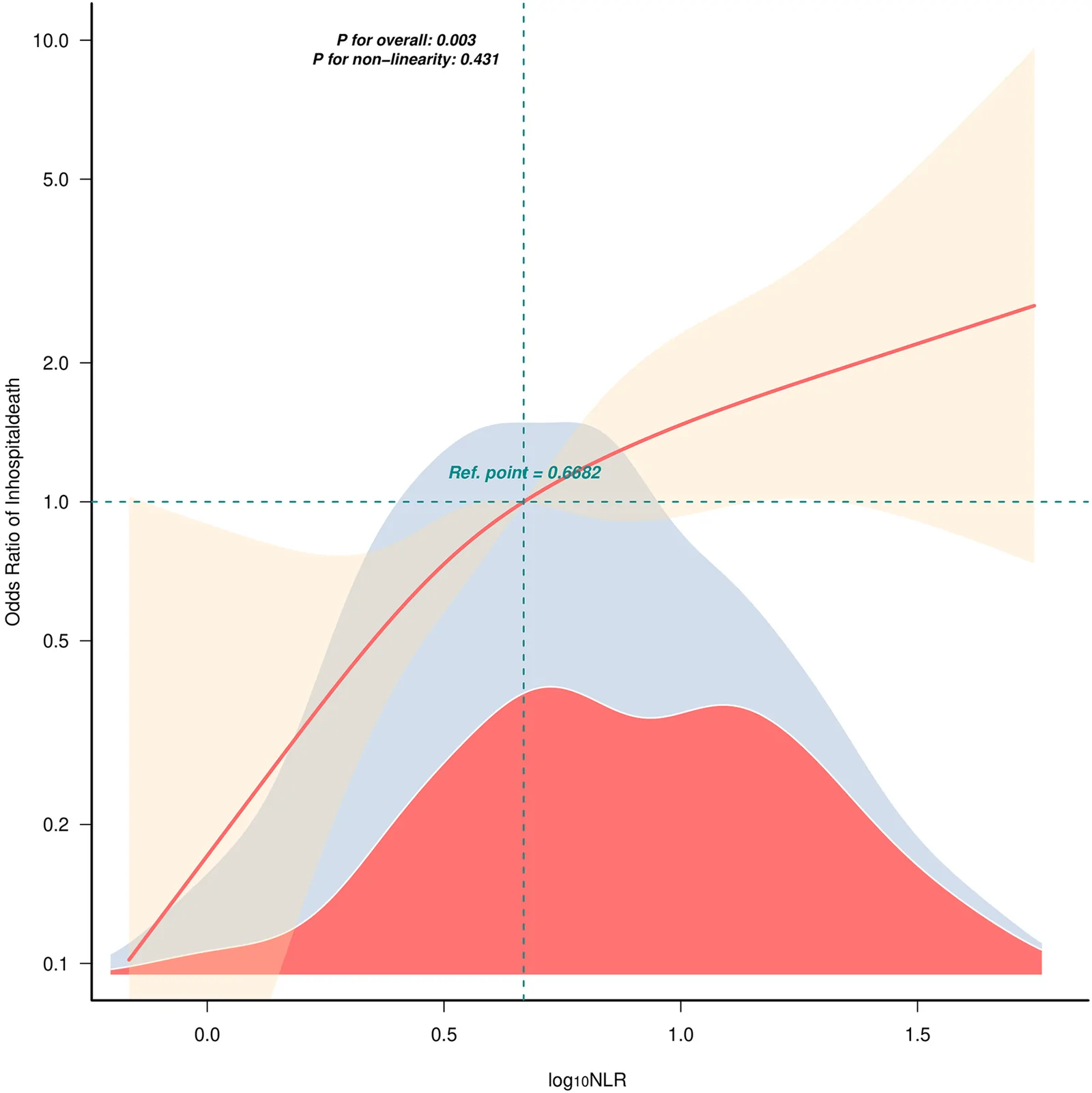
Restricted cubic spline for the association between log_10_NLR and in-hospital mortality in patients with liver cirrhosis complicated by pneumonia. The solid red lines represent the multivariate-adjusted odds ratios derived from restricted cubic spline regression, with the yellow shaded area indicating the 95% confidence interval. The blue and red shaded areas represent the density curves for patients who survived and those who did not survive, respectively. The reference point was set at the median log_10_NLR value of 0.6682 (OR = 1.0, indicated by the vertical dashed line). *P* for overall association = 0.003; *P* for non-linearity = 0.431.

### Sensitivity analyses

Sensitivity analyses were performed to verify the robustness of the primary findings. Supplementary Table S3 presents the main results based on the complete case dataset (*n* = 392). The association between log_10_NLR and in-hospital mortality remained stable (Model 4: OR = 6.67, 95% CI: 2.42–18.39, *P* < 0.001). To further account for patient clustering within individual hospitals, we performed generalized linear mixed-effects logistic regression with hospital-level random intercept as an additional sensitivity test. This model included the same set of confounding covariates as the fully-adjusted Model 4. The random-intercept variance was 1.375, confirming notable inter-hospital heterogeneity of baseline mortality risk. After adjustment, log_10_NLR remained independently associated with in-hospital mortality (adjusted OR = 3.82 95% CI: 1.99–7.34, *P* < 0.001). The association between log_10_NLR and in-hospital mortality remained robust after additional adjustment for calendar time, both when modeled as a categorical period (2016–2021 vs. 2022–2025; adjusted OR: 3.74, 95% CI: 1.92–7.27; *P* < 0.001) and as a continuous year (adjusted OR: 3.49, 95% CI: 1.79–6.81; *P* < 0.001) (Supplementary Table S5).

Forest plot-based subgroup analyses (Figure 3) showed consistent associations between log_10_NLR and in-hospital mortality across all prespecified subgroups—defined by age, sex, albumin concentration, comorbidities (CAD, hypertension, diabetes, prior malignancy, and hepatitis type), severity scores (Child-Pugh and CURB-65), ICU admission, and calendar period (2016–2021 vs. 2022–2025), and participating center—with no significant interaction detected (all *P* for interaction >0.05). Taken together, these clinical variables did not significantly modify the prognostic value of log_10_NLR, supporting the robustness of our main findings.

**Figure 3:**
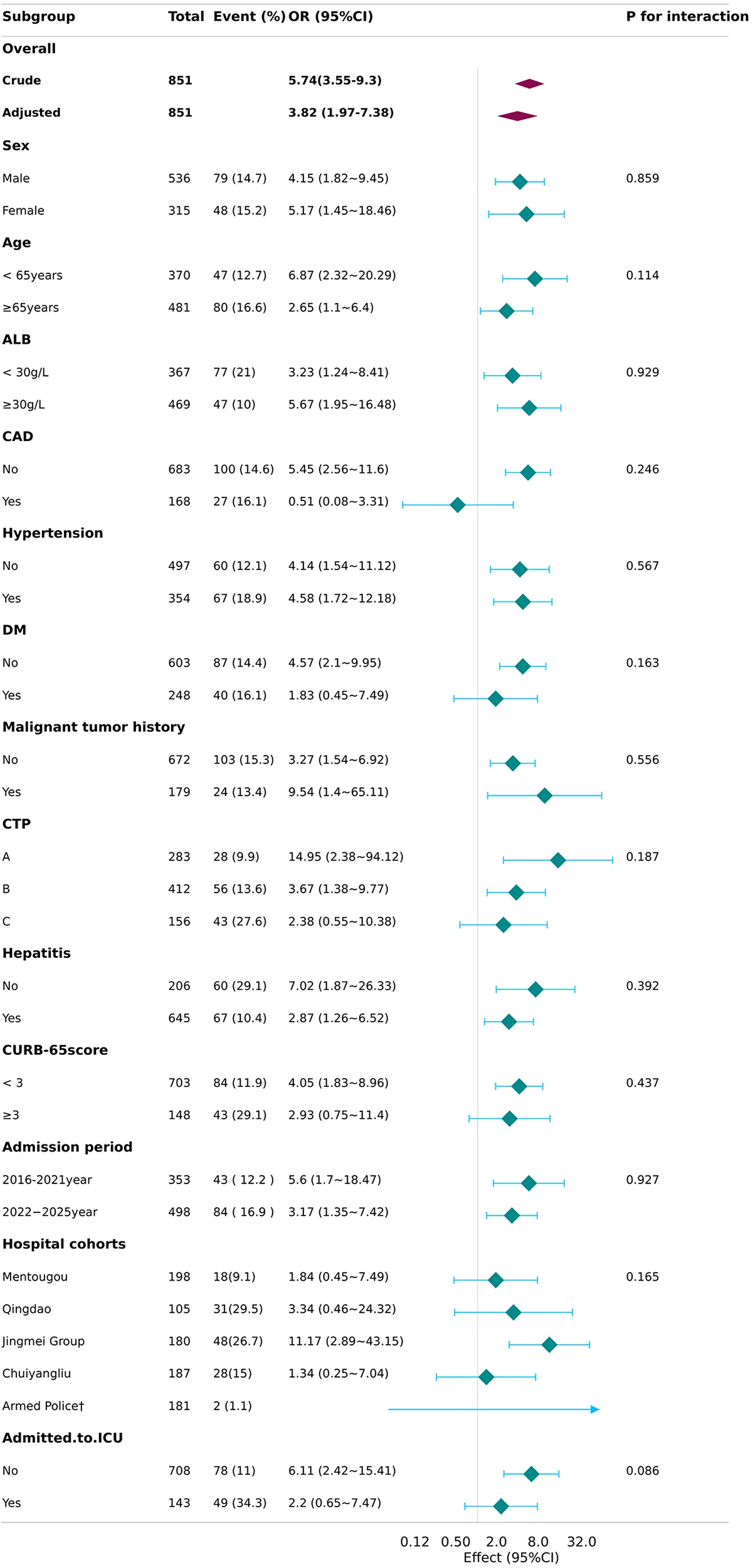
Forest plot of subgroup analyses for the association between log_10_NLR and in-hospital mortality. OR, odds ratio; CI, confidence interval; log_10_NLR, logarithm base 10 of the neutrophil-to-lymphocyte ratio; ALB, albumin; CAD, coronary atherosclerotic heart disease; DM, diabetes mellitus.

### Receiver operating characteristic curve analysis

ROC curves (Figure 4) compared the discriminative performance of log_10_NLR, the Child-Pugh score, and the CURB-65 score for in-hospital mortality. log_10_NLR had an AUC of 0.707 (95% CI: 0.660–0.754), numerically higher than the Child-Pugh (0.647, 95% CI: 0.595–0.699) and CURB-65 scores (0.658, 95% CI: 0.610–0.707), although the differences were not statistically significant (DeLong *P* > 0.05). The Youden-optimal cutoff for log_10_NLR in this cohort was 0.697, corresponding to an NLR of approximately 5 on the original scale, with a sensitivity of 74.0%, a specificity of 57.6%, a PPV of 23.4%, and an NPV of 92.7% (Table 3). In the complete-case dataset, discriminative performance was consistent with the primary analysis (AUC 0.717, 95% CI: 0.652–0.781; Supplementary Figure S3 and Table S6).

**Figure 4:**
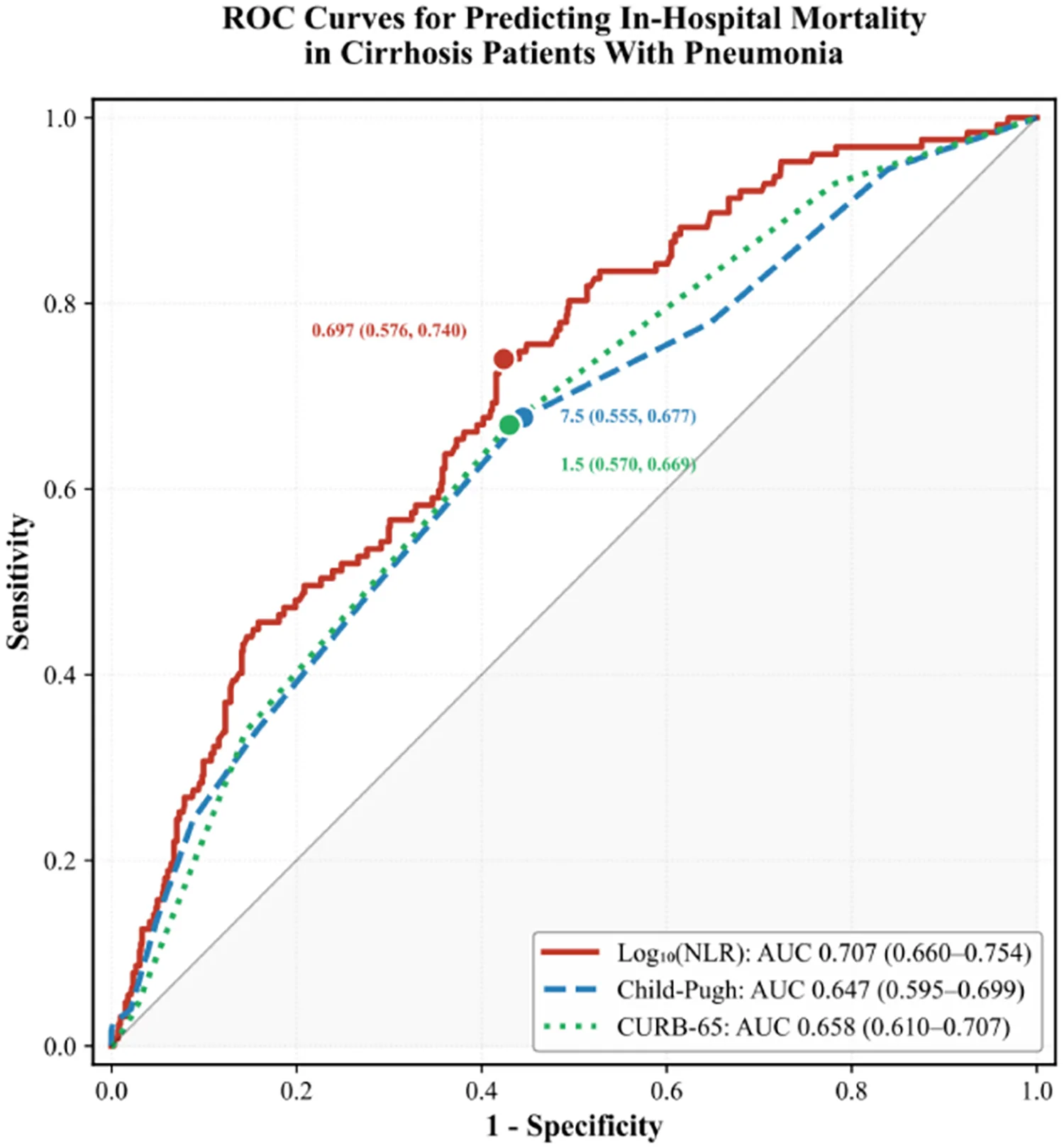
Receiver operating characteristic (ROC) curves comparing the discriminative performance of admission log_10_NLR, Child-Pugh score, and CURB-65 score for in-hospital mortality. The area under the curve (AUC) for log_10_NLR was 0.707 (95% CI: 0.660–0.754), comparable to the Child-Pugh score (AUC: 0.647, 95% CI: 0.595–0.699) and the CURB-65 score (AUC: 0.658, 95% CI: 0.610–0.707). Solid dots indicate the Youden-optimal (exploratory) cutoff point for each model.

**Table 3:** Receiver operating characteristic curve analysis of variables for in-hospital mortality in patients with cirrhosis and pneumonia.

Variable	AUC	95% CI	Cutoff value	NPV	PPV	Sensitivity (%)	Specificity (%)	Youden's index
Log_10_NLR	0.707	0.660–0.754	0.697	0.927	0.234	74.0	57.6	0.316
Child-Pugh	0.647	0.595–0.699	8	0.907	0.211	67.7	55.5	0.232
CURB-65	0.658	0.610–0.707	2	0.908	0.215	66.9	57.0	0.239

AUC, area under the curve; 95% CI, 95% confidence interval; NPV, negative predictive value; PPV, positive predictive value.

Adding log_10_NLR significantly improved discrimination. The AUC increased from 0.647 (95% CI: 0.595–0.699) to 0.737 (95% CI: 0.692–0.782) for Child-Pugh (ΔAUC = 0.090; DeLong *P* < 0.001), and from 0.658 (95% CI: 0.610–0.707) to 0.734 (95% CI: 0.689–0.779) for CURB-65 (ΔAUC = 0.076; *P* < 0.001) (Figures 5A,B; Table 4). Calibration curves showed acceptable agreement between predicted and observed risks (Figures 5C,D). In decision-curve analysis, the models incorporating log_10_NLR provided greater net benefit across clinically relevant threshold probabilities (approximately 10%–40%) (Figures 5E,F).

**Figure 5:**
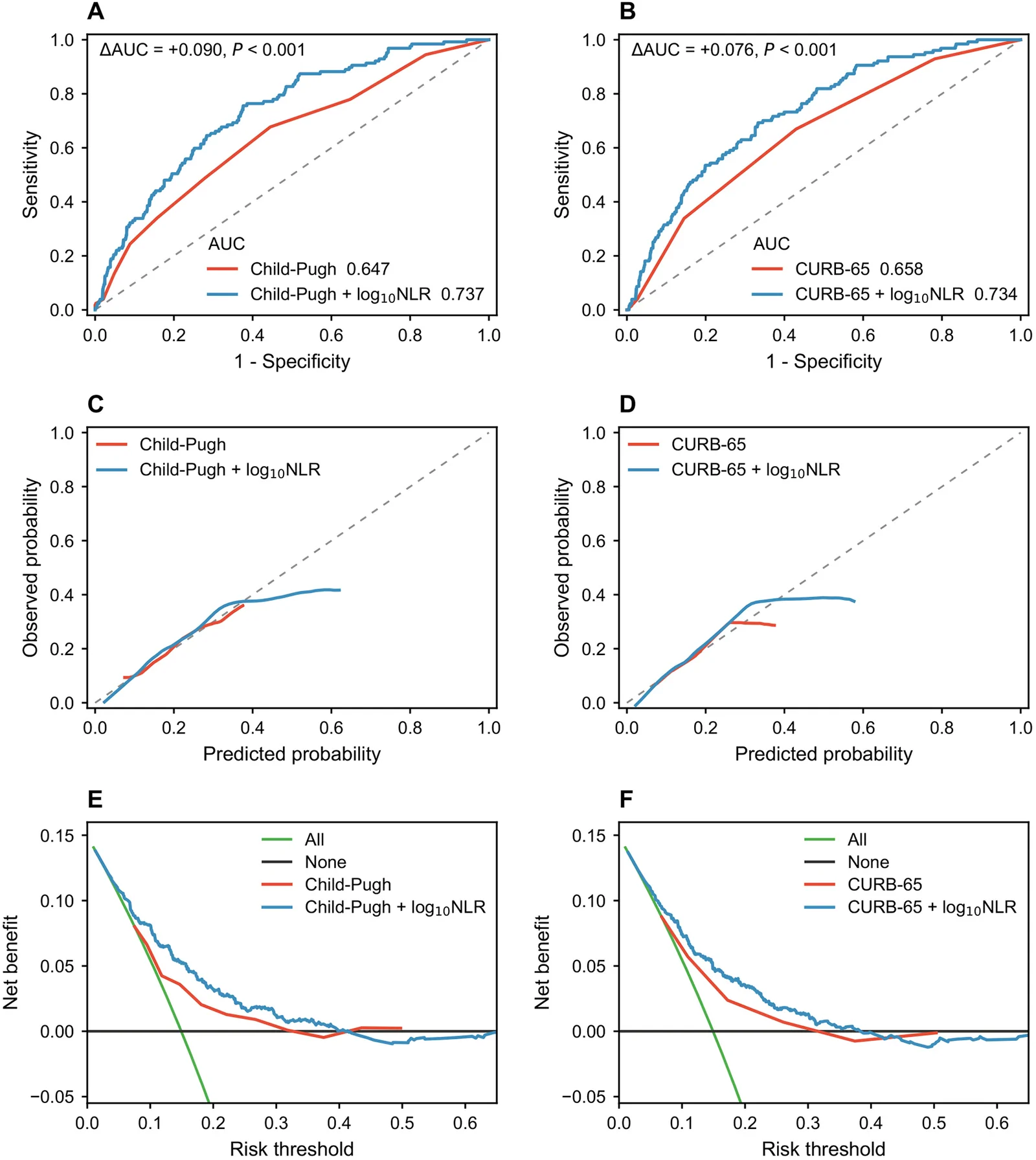
Incremental value of log_10_NLR over established prognostic scores. **(A,B)** ROC curves comparing Child-Pugh **(A)** and CURB-65 **(B)** alone with each score plus log_10_NLR; ΔAUC and DeLong *P* values are shown in each panel. **(C,D)** Calibration curves for models based on Child-Pugh **(C)** and CURB-65 **(D)**. **(E,F)** Decision-curve analyses showing net benefit across threshold probabilities for Child-Pugh **(E)** and CURB-65 **(F)** AUC, area under the receiver operating characteristic curve; NLR, neutrophil-to-lymphocyte ratio.

**Table 4:** Incremental discriminatory value of log_10_NLR added to established prognostic scores.

Model	AUC	95% CI	△AUC	*P* value*
Child-Pugh	0.647	0.595–0.699	—	
Child-Pugh + Log_10_NLR	0.737	0.692–0.782	+0.090	<0.001
CURB-65	0.658	0.610–0.707	—	
CURB-65 + Log_10_NLR	0.734	0.689–0.779	+0.076	<0.001

*n* = 851.

* *P* value from the DeLong test comparing the AUC of each model incorporating log_10_NLR with that of the corresponding baseline score alone.

ΔAUC, increase in AUC after adding log_10_NLR; AUC, area under the receiver operating characteristic curve; CI, confidence interval.

## Discussion

This multicenter retrospective cohort study demonstrated that the NLR is independently associated with in-hospital mortality in patients with cirrhosis and pneumonia. After grouping by NLR tertiles, the mortality rates in the T1, T2, and T3 groups were 5.3%, 15.2%, and 24.3%, respectively (*P* < 0.001) (Table 1). Baseline characteristics indicate that NLR stratification is associated with clinical features: patients in the high NLR group (T3) had more severe liver dysfunction, more severe pneumonia, and a more pronounced systemic inflammatory response, with all differences being statistically significant (all *P* < 0.001). There were no significant differences among the three groups in terms of age, sex, platelet count, or transaminase levels, suggesting that the prognostic value of the NLR is independent of these factors. The multivariate adjusted model showed that an elevated log_10_NLR was independently and robustly associated with increased in-hospital mortality in patients with cirrhosis complicated by pneumonia (Table 2), and sensitivity analyses also confirmed this finding. For every one-unit increase in log_10_NLR (i.e., a 10-fold increase in NLR), the odds of in-hospital death were 3.82-fold higher (OR: 3.82, 95% CI: 1.97–7.38, *P* < 0.001); the odds of death in the T3 group were 3.35 times those in the T1 group (OR: 3.35, 95% CI: 1.65–6.78, *P* = 0.001). Log_10_NLR showed a linear relationship with in-hospital mortality (*P* = 0.003, Figure 2), consistent with previous reports (25). The NLR can identify bacterial infections in patients with acute decompensated cirrhosis and predict short-term mortality; it is positively correlated with CTP, MELD scores, and the severity of ACLF. Other reports (26) have indicated that the NLR is an independent risk factor for all-cause in-hospital mortality in patients with sepsis complicated by cirrhosis.

This study compared the discriminative performance of log_10_NLR with the Child-Pugh and CURB-65 scores for in-hospital mortality in patients with cirrhosis complicated by pneumonia and found that log_10_NLR (AUC: 0.707, 95% CI: 0.660–0.754) demonstrated comparable discriminative ability to the other two scores. Importantly, adding log_10_NLR to these scores significantly improved discrimination (ΔAUC = 0.090 for Child-Pugh and 0.076 for CURB-65; both DeLong *P* < 0.001) and increased net benefit in decision-curve analysis across clinically relevant thresholds, suggesting potential clinical usefulness. However, because these models were derived and evaluated in the same cohort, external validation is required before clinical implementation. Consistent with previous studies (27), the NLR is an independent predictor of mortality in patients with cirrhosis, independent of CTP and MELD scores. Janka et al. (25) also found that NLR outperformed white blood cell count in identifying bacterial infections and predicting short-term mortality in patients with acute decompensated cirrhosis; in patients without ACLF, it even replaced the white blood cell count in the CLIF-CAD score. In this population, Xu et al. (28) compared the predictive value of six scoring systems (CTP, MELD, PSI, qSOFA, CLIF-SOFA) for 30-day and 90-day mortality and found that CLIF-SOFA performed best (30-day AUROC: 0.89, 90-day AUROC: 0.90); however, it requires assessment of six organ systems and is primarily suitable for use in the ICU. The ROC curve indicates that the exploratory cutoff value for log_10_NLR is 0.697 (corresponding to an NLR of approximately 5), with a Child-Pugh score ≥8 (Class B/C) and a CURB-65 score ≥2 (moderate-to-severe pneumonia). This suggests that the risk of death is significantly elevated when patients have CTP class B/C liver function, moderate-to-severe pneumonia (CURB-65 ≥ 2), and an NLR ≥ 5. Therefore, an NLR ≥ 5 may serve as an exploratory cutoff for identifying patients at higher risk of in-hospital death, though this requires validation in independent cohorts. Chiriac et al. (29) reported that the AUC of the NLR for predicting mortality in patients with ACLF was 0.776, with an optimal cutoff value of 5; Janka et al. (25) found that when the NLR was >5.32, the likelihood of bacterial infection was increased in patients with acute decompensated cirrhosis.

Immune dysfunction in cirrhosis is characterized by systemic inflammation and immunodeficiency; its severity varies depending on the stage of cirrhosis and the presence of acute events such as infections. Typical manifestations include elevated markers of neutrophil activation and suppressed lymphocyte function (2). Following the onset of pneumonia, neutrophils are activated through various mechanisms (30), leading to a further increase in the NLR. Elevated NLR levels reflect both the intensity of the body's immune response to pneumonia and the severity of immune dysfunction associated with cirrhosis. As the disease progresses, the balance between pro-inflammatory and anti-inflammatory responses gradually becomes disrupted (31). Neutrophilia suggests infection and systemic inflammation driven by endotoxemia, while lymphopenia reflects immune exhaustion and an increased risk of secondary infection (32). Therefore, the NLR can comprehensively assess a patient's inflammatory burden and immune status. Multiple studies have confirmed (33, 34) that the NLR is a prognostic marker in patients with cirrhosis, and its predictive value for short-term mortality risk is superior to its value for long-term prognosis assessment. An elevated NLR is positively correlated with the risk of infection (31); it has been reported that for every 1-unit increase in the NLR, the risk of infection increases by 1.29 times (35), suggesting that the NLR can serve as a marker for stratifying the risk of infection in patients with cirrhosis. Studies by Kwon et al. (36) and Liu et al. (37) both found that, among patients with cirrhosis in the same CTP grade, those with concurrent infections had higher NLR levels and a poorer prognosis. In this study, the NLR showed a weak positive correlation with the Child-Pugh score (*r* = 0.203, *P* < 0.001) and the CURB-65 score (*r* = 0.283, *P* < 0.001), suggesting that an elevated NLR is associated with disease severity, consistent with previous reports in the literature (14, 38). The prognostic value of the NLR in patients with pneumonia has been widely reported. One retrospective study showed (39) that an elevated NLR is associated with an increased risk of ICU admission in patients with community-acquired pneumonia. Another study indicated (40) that the combination of the National Early Warning Score (NEWS) and the NLR is more effective than NEWS alone in assessing the prognosis of elderly patients with pneumonia. A report by Turbawaty et al. (41) suggests that the NLR on the day of diagnosis of ventilator-associated pneumonia is a predictor of in-hospital mortality. Pneumonia is a major trigger for the development of ACLF in patients with cirrhosis (42). As a convenient biomarker that takes both inflammation and immunity into account, the NLR can serve as a complementary tool to existing disease severity scoring systems.

The clinical management of patients with cirrhosis complicated by pneumonia is complex. Due to impaired immune function, these patients are prone to infections, and the inflammatory response triggered by pneumonia further exacerbates this immune imbalance. At the same time, patients with cirrhosis have reduced synthesis of coagulation factors, and infection activates the coagulation system, leading to an increased risk of both bleeding and thrombosis. Oxygenation impairment caused by pneumonia can accelerate the deterioration of liver function and may even trigger multi-organ dysfunction, such as hepatorenal syndrome. Furthermore, impaired liver and kidney function limits the selection and use of antibiotics. Therefore, early identification and risk stratification of high-risk patients with cirrhosis complicated by pneumonia are crucial for improving prognosis.

In this study, log_10_NLR was independently associated with in-hospital mortality and added incremental discriminatory value beyond the Child-Pugh and CURB-65 scores. Because it is derived from routine blood counts and requires no additional testing or scoring, log_10_NLR may be particularly useful for early risk screening in settings where more complex severity scores are not routinely calculated.

This study has several limitations. First, despite its multicenter design, the retrospective nature may introduce residual confounding, and prospective validation is warranted. Second, only baseline log_10_NLR at admission was measured, and dynamic changes were not monitored, which might provide additional prognostic information; moreover, NLR levels may be influenced by stress and glucocorticoid use. Third, the specific etiology of pneumonia was not identified, and the analysis was not stratified by cirrhosis etiology. Fourth, although we adjusted for participating center and calendar time (with consistent results in a mixed-effects model and time-sensitivity analyses), residual confounding by unmeasured center-level factors (e.g., ICU availability, treatment protocols, and discharge policies) and by temporal changes in clinical practice cannot be fully excluded. Fifth, in-hospital mortality was used as the endpoint; future studies should evaluate 30-day and 90-day prognosis.

## Conclusion

Log_10_NLR was independently associated with in-hospital mortality in cirrhosis complicated by pneumonia in a linear dose–response manner. Adding it to the Child-Pugh and CURB-65 scores significantly improved discrimination and net benefit, supporting its potential as a simple, low-cost complementary marker. Prospective, multicenter validation is warranted.

## Data Availability

The data that support the findings of this study are available from the corresponding author upon reasonable request. The raw data are not publicly accessible owing to patient privacy and ethical restrictions.
