# Supplementary material for: Neutrophil-to-lymphocyte ratio is associated with in-hospital mortality in patients with cirrhosis and pneumonia: a multicenter retrospective study
Source: Front Med (Lausanne). 2026 Oct 19;13:1963013. doi: 10.3389/fmed.2026.1963013 (PMC13724411; doi:10.3389/fmed.2026.1963013)
Supplement: Supplementary file 1 [file Datasheet1.pdf]

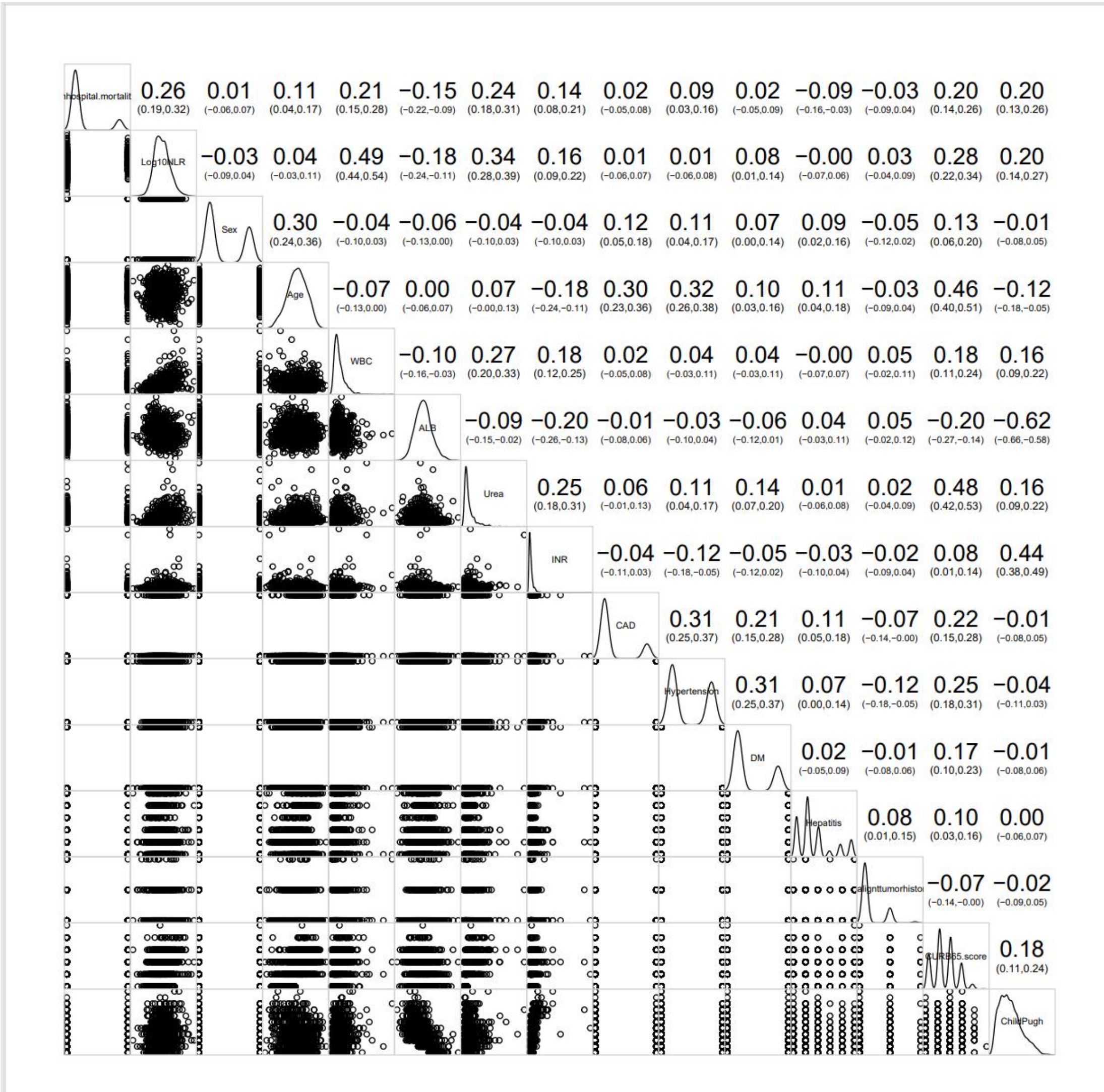

Supplementary Figure 1: Correlation matrix plot of candidate predictors. The diagonal panels show kerne l density estimates for each variable. The lower triangle displays pairwise scatterplots. The upper triangle pr esents Pearson correlation coefficients with 95% confidence intervals (in parentheses). Abbreviations: $\log_{10}$ NLR, neutrophil-to-lymphocyte ratio in log10 scale; CTP: Child-Turcotte-Pugh score; CURB65: Confusion, Urea, Respiratory rate, Blood pressure, age ≥65; WBC:white blood cell count; ALB: albumin; INR:internat ional normalized ratio; CAD: coronary artery disease; DM: diabetes mellitus.

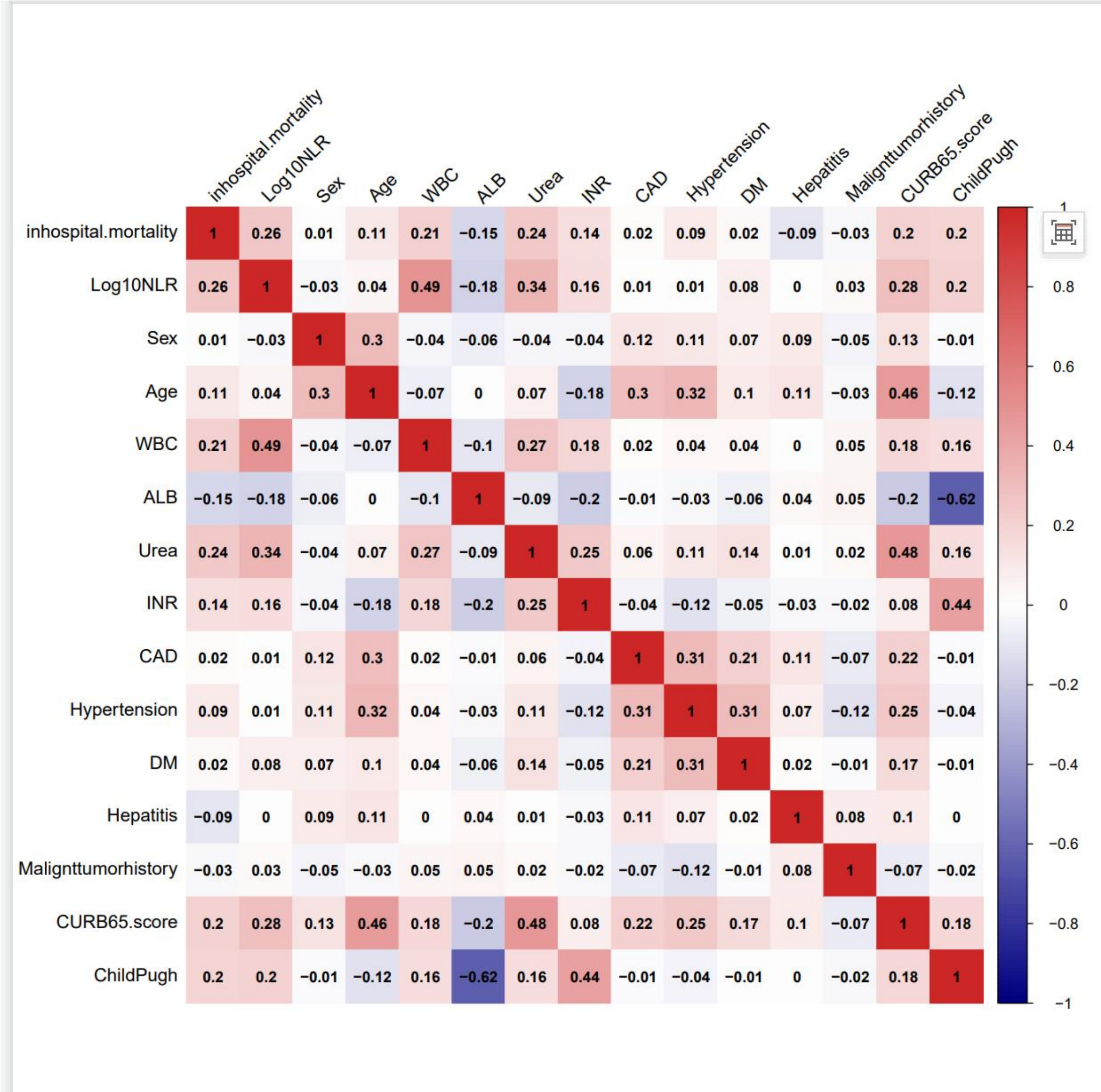

Supplementary Figure 2: Heatmap of Pearson correlation coefficients among candidate predictors. Colors range from blue (negative correlation) to red (positive correlation), with the intensity reflecting the strength of correlation. Abbreviations: $\log_{10}$NLR: neutrophil-to-lymphocyte ratio in log10 scale; CTP: Child-Turcotte-Pugh score; CURB65: Confusion, Urea, Respiratory rate, Blood pressure, age ≥65; WBC:white blood cell count; ALB: albumin; INR:international normalized ratio; CAD: coronary artery disease; DM: diabetes mellitus.

Supplementary Table 1 The variable distribution of the missing data.

Variable	Missing data	Percentage of missing data
Age (years)	0	0
ALB(g/L)	15	1.7626
ALT(U/L)	5	0.5875
APTT(s)	33	3.8778
Ascites	224	26.322
AST(U/L)	15	1.7626
CAD	11	1.2926
Cr(μmol/L)	10	1.1751
DBIL(μmol/L)	29	3.4078
D-dimer(mg/L)	154	18.0964
DM	0	0
Fibrinogen (g/L)	42	4.9354
Hb(g/L)	4	0.47
HeartFailure	1	0.1175
HepaticEncephalopathyHE	223	26.2045
Hepatitis	14	1.6451
Hypertension	1	0.1175
Inhospitaldeath	0	0
LYMR($\times10^9$/L)	0	0
Malignant tumor history	5	0.5875
NEUT($\times10^9$/L)	0	0
PLT($\times10^9$/L)	0	0
PT(s)	33	3.8778
INR	34	3.9953

Sex	1	0.1175
TBIL(μmol/L)	16	1.8801
Urea(mmol/L)	13	1.5276
WBC($\times 10^9$/L)	0	0
Disturbance of consciousness	320	37.6028
RR(br/min)	1	0.1175
SBP(mmHg)	0	0
DBP(mmHg)	0	0
Hospital cohorts	0	0
Admitted to ICU	21	2.4677

Abbreviations: WBC: white blood cell count;PLT:platelet count;Hb:hemoglobin;ALT: alanine ainotransferase;AST: aspartate aminotransferase;TBIL:Totalbilirubin; DBIL: direct bilirubin;ALB:albumin;Cr: serum creatinine;PT: prothrombin time;APTT: activated partial thromboplastin time;INR: international normalized ratio;CAD: Coronary Atherosclerotic Heart Disease; DM: Diabetes Mellitus;NEUT:neutrophil;LYMR:lymphocyte;RR:respiratory rate;SBP:systolic blood pressure;DBP:diastolic blood pressure

Supplementary Table 2. Multivariate logistic regression analysis of the $\log_{10}$NLR and in-hospital mortality in cirrhotic patients with pneumonia (n =851, 40 imputations)

Variable	Crude		Model1		Model2		Model3		Model4	
	OR (95%CI)	P value	OR (95%CI)	P value	OR (95%CI)	P value	OR (95%CI)	P value	OR (95%CI)	P value
$\log_{10}$NLR	5.74(3.55–9.3)	<0.001	5.61(3.45–9.11)	<0.001	7.63 (4.4–13.23)	<0.001	4.91 (2.75–8.77)	<0.001	3.8 (1.96–7.35)	<0.001
$\log_{10}$NLR										
T1(≤0.495)	1(Ref)		1(Ref)		1(Ref)		1(Ref)		1(Ref)	

T2(0.495 to ≤ 0.860)	3.21(1.74–5.93)	<0.001	3.22(1.74–5.95)	<0.001	3.49 (1.83–6.67)	<0.001	3.56 (1.81–7)	<0.001	2.86 (1.47–5.56)	0.002
T3(>0.860)	5.76(3.2–10.35)	<0.001	5.76 (3.2–10.38)	<0.001	7.35 (3.92–13.79)	<0.001	4.96 (2.55–9.66)	<0.001	3.33 (1.65–6.75)	0.001
P for trend		<0.001		<0.001		<0.001		<0.001		0.001

Model1: adjusted for age, sex

Model2: adjusted for age, sex, CAD, hypertension, DM, hepatitis types,hospital cohorts

Model3:adjusted for age, sex, CAD, hypertension, DM, hepatitis types ,hospital cohorts,Child-Pugh,CURB-65

Model4: adjusted for age, sex, CAD, hypertension, DM, hepatitis types, hospital cohorts,Urea,WBC,INR, ALB

Abbreviations: OR: odds ratio; CI: confidence interval; log_{10}NLR: logarithm base 10 of the neutrophil-to- lymphocyte ratio; CAD: Coronary Atherosclerotic Heart Disease; DM: Diabetes Mellitus;WBC:white blood cell count;ALB:albumin;INR: i nternational normalized ratio

Supplementary Table 3 Multivariate logistic regression analysis of the log_{10}NLR and in-hospital mortality in cirrhotic patients with pneumonia (excluding missing values, n =392)

Variable	Crude		Model1		Model2		Model3		Model4	
	OR (95%CI)	P value	OR (95%CI)	P value	OR (95%CI)	P value	OR (95%CI)	P value	OR (95%CI)	P value
log_{10}NLR	7.86 (3.84~16.11)	<0.001	7.68 (3.72~15.83)	<0.001	10.18 (4.5~23.02)	<0.001	6.09 (2.61~14.22)	<0.001	6.67 (2.42~18.39)	<0.001
log_{10}NLR										
T1(≤ 0.495)	1(Ref)		1(Ref)		1(Ref)		1(Ref)		1(Ref)	
T2(0.495 to ≤ 0.860)	3.07 (1.37~6.89)	0.007	3.3 (1.46~7.46)	0.004	3.53 (1.51~8.28)	0.004	4.12 (1.63~10.37)	0.003	3.11 (1.28~7.59)	0.012
T3(>0.860)	5.75 (2.65~12.46)	<0.001	6.07 (2.78~13.25)	<0.001	7.45 (3.24~17.13)	<0.001	5.45 (2.23~13.35)	<0.001	4.18 (1.6~10.92)	0.004
P for trend		<0.001		<0.001		<0.001		<0.001		0.004

Model1: adjusted for age, sex

Model2: adjusted for age, sex, CAD, hypertension, DM, hepatitis types,hospital cohorts,

Model3: adjusted for age, sex, CAD, hypertension, DM, hepatitis types, hospital cohorts, Child-Pugh , CURB-65

Model4: adjusted for age, sex, CAD, hypertension, DM, hepatitis types,hospital cohorts,Urea,WBC,INR, ALB

Abbreviations: OR: odds ratio; CI: confidence interval; log_{10}NLR: logarithm base 10 of the neutrophil-to- lymphocyte ratio; CAD: Coronary Atherosclerotic Heart Disease; DM: Diabetes Mellitus;WBC:white blood cell count;ALB:albumin;INR: international normalized ratio

Supplementary Table 4. Patients, in-hospital mortality, and center-specific random intercepts by participating center

Center	Patients, n (%)	Survivors (n = 724)	Non-survivors (n = 127)	Mortality rate, %	Random intercept estimate
Mentougou	198 (23.3)	180 (24.9)	18 (14.2)	9.1	−0.03
Armed Police	181 (21.3)	179 (24.7)	2 (1.6)	1.1	−1.92
Qingdao	105 (12.3)	74 (10.2)	31 (24.4)	29.5	+1.29
Jingmei Group	180 (21.2)	132 (18.2)	48 (37.8)	26.7	+0.70
Chuiyangliu	187 (22.0)	159 (22.0)	28 (22.0)	15.0	+0.31
Total	851 (100.0)	724 (100.0)	127 (100.0)	14.9	

Notes: Data are n (%). P <0.001 across the five centers (χ^2 test). Mortality rate = non-survivors / patients within each center.Random-intercept variance 1.375; ICC 0.29. Random intercepts are center-specific deviations from the overall intercept on the log-odds scale; they are consistent with the observed mortality gradient across centers.

Supplementary Table 5. Sensitivity analyses of the association between $\log_{10}$NLR and in-hospital mortality.

Model	Adjusted OR (95% CI) for $\log_{10}$NLR	P
Primary (fully adjusted Model 4)	3.82 (1.97–7.38)	<0.001
Mixed-effects model (hospital random intercept)	3.82 (1.99–7.34)	<0.001
+ admission period (categorical)	3.74 (1.92–7.27)	<0.001
+ admission year (continuous)	3.49 (1.79–6.81)	<0.001

Notes: All models were adjusted for the same covariates as the fully adjusted Model 4. The mixed-effects model included a hospital-level random intercept (random-intercept variance 1.375; ICC = 0.29). Admission period was categorized as 2016–2021 and 2022–2025. n = 851; 127 in-hospital deaths (14.9%). NLR, neutrophil-to-lymphocyte ratio; OR, odds ratio; CI, confidence interval; ICC, intraclass correlation coefficient.

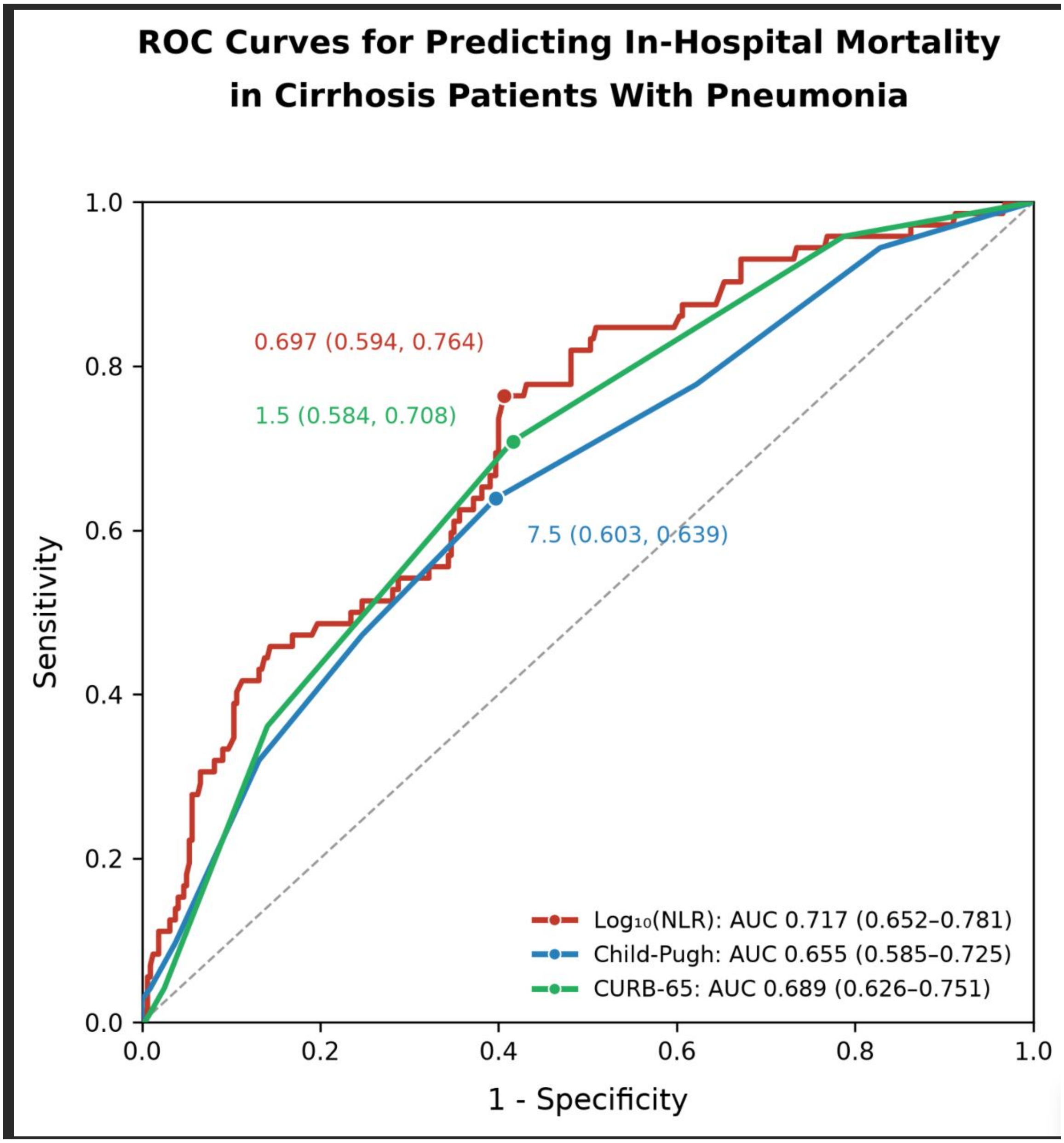

Supplementary Figure 3. Receiver operating characteristic curves of log_{10}NLR, Child-Pugh score, and CURB-65 score for predicting in-hospital mortality in the complete case dataset (n = 392). Solid dots indicate the optimal cutoff values determined by the Youden index. Values in parentheses next to each dot represent (specificity, sensitivity) at the optimal cutoff.

Supplementary Table 6. Receiver operating characteristic curve analysis of variables for in-hospital mortality in patients with cirrhosis and pneumonia

Variable	AUC	95% CI	Cutoff value	NPV	PPV	Sensitivity (%)	Specificity (%)	Youden index
log_{10}NLR	0.717	0.652–0.781	0.697	0.918	0.297	76.4	59.4	0.358
Child-Pugh	0.655	0.585–0.725	8	0.881	0.266	63.9	60.3	0.242
CURB-65	0.689	0.626–0.751	2	0.899	0.277	70.8	58.4	0.293

Abbreviations: AUC: area under the curve; NPV: negative predictive value; PPV: positive predictive value.

Supplementary Table 7. Comparison of patients with complete data and patients with missing data

Variables	Total (n = 851)	Complete data (n = 392)	Missing data (n = 459)	P value
Age (years)b	66.5 ± 12.7	68.3 ± 12.5	65.0 ± 12.7	< 0.001
WBC (×10^9/L)c	5.5 (3.7–8.5)	5.9 (4.0–8.7)	5.4 (3.6–8.4)	0.068
Hemoglobin (g/L)b	101.9 ± 29.6	103.2 ± 28.4	100.8 ± 30.7	0.229
Platelet count (×10^9/L)c	98.0 (64.0–148.0)	105.0 (69.0–157.0)	93.0 (60.0–138.5)	0.006
ALT (U/L)c	21.7 (14.0–35.0)	21.5 (14.0–34.0)	22.0 (14.0–36.0)	0.650
AST (U/L)c	34.0 (22.4–55.0)	34.0 (22.4–54.0)	34.0 (22.3–57.2)	0.865
Total bilirubin (μmol/L)c	22.9 (13.6–42.1)	22.4 (13.5–41.5)	23.1 (13.7–43.1)	0.450
Direct bilirubin (μmol/L)c	10.3 (5.6–22.0)	10.1 (5.3–20.8)	10.8 (6.1–22.4)	0.128
Albumin (g/L)b	31.1 ± 5.9	30.7 ± 5.7	31.4 ± 6.2	0.066
Urea (mmol/L)c	6.8 (4.8–11.0)	6.9 (5.0–10.8)	6.7 (4.6–11.2)	0.446
Creatinine (μmol/L)c	72.0 (55.0–102.0)	69.5 (54.0–101.2)	74.0 (56.0–102.0)	0.219
Respiratory rate (breaths/min)c	20.0 (18.0–20.0)	20.0 (18.0–20.0)	20.0 (20.0–20.0)	0.009
Prothrombin time	14.8 (13.2–17.1)	14.2 (12.6–16.4)	15.3 (13.7–17.4)	< 0.001

(s)c				
APTT (s)c	36.5 (31.5–41.5)	34.5 (30.7–39.4)	38.3 (33.4–43.2)	< 0.001
INRc	1.2 (1.1–1.4)	1.2 (1.1–1.4)	1.2 (1.1–1.5)	0.005
D-dimer (mg/L)c	2.0 (0.7–5.0)	2.1 (0.7–5.7)	1.8 (0.6–4.2)	0.055
CURB-65 scorec	1.0 (1.0–2.0)	1.0 (1.0–2.0)	1.0 (1.0–2.0)	0.381
$\log_{10}$NLRc	0.7 (0.4–0.9)	0.7 (0.4–1.0)	0.7 (0.4–0.9)	0.869
Malea	314 (36.9)	142 (36.2)	172 (37.5)	0.760
CADa	167 (19.6)	77 (19.6)	90 (19.6)	1.000
Hypertensiona	354 (41.6)	184 (46.9)	170 (37.0)	0.004
Diabetes mellitusa	248 (29.1)	107 (27.3)	141 (30.7)	0.308
Heart failurea	64 (7.5)	36 (9.2)	28 (6.1)	0.116
Prior malignancya	166 (19.5)	86 (21.9)	80 (17.4)	0.117
Hepatitis				< 0.001
None	201 (24.0)	125 (31.9)	76 (17.1)	
Viral	303 (36.2)	120 (30.6)	183 (41.1)	
Alcoholic	151 (18.0)	53 (13.5)	98 (22.0)	
Others	182 (21.7)	94 (24.0)	88 (19.8)	
In-hospital mortalitya	127 (14.9)	72 (18.4)	55 (12.0)	0.012
Ascites				0.015
Grade 1	325 (51.8)	216 (55.1)	109 (46.4)	
Grade 2	130 (20.7)	84 (21.4)	46 (19.6)	
Grade 3	172 (27.4)	92 (23.5)	80 (34.0)	
Hepatic encephalopathy				< 0.001
Grade 1	550 (87.6)	360 (91.8)	190 (80.5)	
Grade 2	64 (10.2)	26 (6.6)	38 (16.1)	
Grade 3	14 (2.2)	6 (1.5)	8 (3.4)	
Disturbance of consciousness	531	392	139	0.929

Notes: Data are presented as mean ± SD, median (IQR), or n (%). a χ^2 test; b Student's t-test; c Mann–Whitney U test; d Fisher's exact test. "Complete data" denotes patients with no missing values for any of the baseline variables listed in Table 1; the remaining patients were classified as "missing data". For variables with missing values, the "Total" column and the group rows showing "Total available" refer to patients in whom the variable was recorded; ascites and hepatic encephalopathy were graded 1–3, and hepatitis aetiology was classified as none, viral, alcoholic, or others. P values for graded variables refer to the overall distribution across categories. Fibrinogen was recorded in inconsistent units across centres and was therefore used only as an auxiliary variable in the imputation model, not as an analysis variable. Variables with no missing values (age, sex, WBC, neutrophil and lymphocyte counts, platelet count, diabetes mellitus, hospital cohort, systolic and diastolic blood pressure, and in-hospital mortality) are shown for completeness; their distributions are identical by definition. CURB-65, Confusion, Urea, Respiratory rate, Blood pressure, and age ≥ 65 years; SD, standard deviation; IQR, interquartile range.
